# 2D and 3D Self-Assembling Nanofiber Hydrogels for Cardiomyocyte Culture

**DOI:** 10.1155/2013/285678

**Published:** 2012-12-31

**Authors:** Liisa Ikonen, Erja Kerkelä, Gerald Metselaar, Marc C. A. Stuart, Menno R. de Jong, Katriina Aalto-Setälä

**Affiliations:** ^1^Institute of Biomedical Technology (IBT), University of Tampere, Biokatu 12, 33520 Tampere, Finland; ^2^BioMediTech, Biokatu 10, 33520 Tampere, Finland; ^3^Finnish Red Cross Blood Service, Kivihaantie 7, 00310 Helsinki, Finland; ^4^Nano Fiber Matrices B.V., Nijenborgh 4, 9747 AG Groningen, The Netherlands; ^5^BASF Nederland B.V., Innovatielaan 1, 8466 SN Heerenveen, The Netherlands; ^6^Department of Biophysical Chemistry, University of Groningen, Nijenborgh 7, 9747 AG Groningen, The Netherlands; ^7^Heart Center, Tampere University Hospital, Teiskontie 35, 33521 Tampere, Finland

## Abstract

Collagen is a widely used biomaterial in cardiac tissue engineering studies. However, as a natural material, it suffers from variability between batches that can complicate the standardization of culture conditions. In contrast, synthetic materials are modifiable, have well-defined structures and more homogeneous batches can be produced. In this study, several collagen-like synthetic self-assembling nanofiber hydrogels were examined for their suitability for cardiomyocyte culture in 2D and 3D. Six different nanofiber coatings were used in the 2D format with neonatal rat cardiomyocytes (NRCs) and human embryonic stem-cell-derived cardiomyocytes (hESC-CMs). The viability, growth, and functionality of the 2D-cultured cardiomyocytes were evaluated. The best-performing nanofiber coatings were selected for 3D experiments. Hydrophilic pH-sensitive nanofiber hydrogel coassembled with hyaluronic acid performed best with both NRCs and hESC-CMs. Hydrophilic non-pH-sensitive nanofiber hydrogels supported the growth of NRCs; however, their ability to promote attachment and growth of hESC-CMs was limited. NRCs also grew on hydrophobic nanofiber hydrogels; however, the cell-supporting capacity of these hydrogels was inferior to that of the hydrophilic hydrogel materials. This is the first study demonstrating that hydrophilic self-assembling nanofiber hydrogels support the culture of both NRCs and hESC-CMs, which suggests that these biomaterials hold promise for cardiac tissue engineering.

## 1. Introduction

Heart failure arising from myocardial loss is a leading cause of morbidity and mortality worldwide [1] for which stem cell therapy is an emerging treatment option. For treatment of heart failure, cells can potentially be delivered to the site of injury where they can repopulate the injured area, integrate into the host tissue, and restore functionality to the myocardium. However, clinical studies have shown that cells delivered by direct injection into the myocardium or by intracoronary injection are rapidly lost [2–4]. Modest improvements in myocardial function have been suggested to merely arise from paracrine effects [5–7]. To achieve the desired cellular effects in addition to the paracrine effects, biomaterial scaffolds can be used to maintain the cells at the site of injection.

An ideal biomaterial scaffold for cardiac repair would allow cardiomyocytes to be grown *in vitro* in a 3D structure that is optimal for application to the heart, allows cellular differentiation, and integrates well into the host tissue after implantation. In addition to clinical cell therapy applications, 3D structures can also provide better cardiac tissue models for studying the pathophysiology of cardiac diseases and the function of diseased cardiomyocytes *in vitro*. Additionally, these 3D models may provide more meaningful physiological data in drug discovery and toxicology assays.

Beating cardiac constructs have been obtained using collagen patches [8], poly(*N*-isopropylacrylamide) sheets [9], collagen rings [10], collagen sponges [11], and injectable fibrin gels [12]. Scaffold-free human cardiac tissue has also been generated [13]. In our previous studies, we compared different synthetic and natural biomaterials for their ability to support cardiomyocyte growth [14]. Of the biomaterials we tested, natural collagen supported cardiomyocyte growth the best but showed significant batch-to-batch variation. Thus, we continued to search for synthetic alternatives to collagen that had similar bioactivity but less variability.

Previously, we tested the commercially available amphiphilic self-assembling peptide PuraMatrix (BD Biosciences). Although PuraMatrix was shown to be less effective than collagen at supporting the growth and survival of cardiomyocytes, it performed well enough to attract our interest in this class of materials [14]. We previously reported the development of self-assembling nanofiber systems with a modular architecture based on a 1,3,5-triamide cis,cis-cyclohexane core [15]. This core functions as a generic nanofiber-forming scaffold that can be easily functionalized and tuned [15–17]. We demonstrated that vesicles can be easily entrapped and immobilized in hydrogels generated from these materials [18], thereby providing a system to study the activity of membrane proteins at the single vesicle level [19]. Preliminary *in vitro* and *in vivo* experiments indicated that hydrogels formed from these compounds are biocompatible [15, 20], which encouraged us to study their use as coatings and hydrogel scaffold materials for the growth of cardiomyocytes.

The aim of this study was to screen different self-assembling nanofiber hydrogels as 2D nanofiber coatings for cardiomyocyte attachment and growth and to then further evaluate the best candidates for use as 3D nanofiber hydrogels. To assess the performance of our nanofiber materials we used NRCs and hESC-CMs, and we considered that an optimal biomaterial should support routine cardiomyocyte attachment, growth, and function. NRCs were used because they can be obtained fairly easily in large numbers, which enabled us to perform large-scale comparison experiments [21] before testing hESC-CMs. It has also been shown that 3D heart tissue-like structures can be created with NRCs [10, 22] and with hESC-CMs [22–24].

Three different types of self-assembling nanofiber hydrogels were examined: hydrophilic pH-sensitive, hydrophilic non-pH-sensitive, and very hydrophobic non-pH-sensitive, hydrogels. We also tested the effect of fiber thickness on cell adhesion for the hydrophilic nanofiber hydrogels. In addition, we tested the effects of pH-sensitive nanofiber hydrogels coassembled with hyaluronic acid (HyA) [25] on cardiomyocyte growth.

## 2. Materials and Methods

### 2.1. Cells

#### 2.1.1. Isolation and Culture of Neonatal Rat Cardiomyocytes

NRCs were isolated as described previously [26], with minor modifications. Briefly, rat hearts were harvested from one- to three-day-old Sprague Dawley rats and then disaggregated in a collagenase (type 2, Worthington, USA) solution. The cells were then preplated to allow the nonmyocytes to attach. The unattached myocytes were collected, counted, and plated onto various nanofiber materials in culture medium I (CMI, Dulbecco's Modified Eagle's Medium/Ham's Nutrient Mixture F-12 (DMEM/F-12, Sigma-Aldrich, USA), 10% fetal bovine serum (FBS, Invitrogen, USA), 100 IU/mL penicillin/0.1 mg/mL streptomycin (P/S, Lonza, Belgium), and 2.56 mM L-glutamine (Sigma-Aldrich)). The cells were plated at equal numbers on nanofiber test materials and uncoated commercial 24-well plates (Nunc, Thermo Fisher Scientific, USA) (400,000 cells/well for 2D coatings or 800,000 inside and on top of the gels). The following day and subsequently every second or third day thereafter, serum-free medium (SFM, DMEM/F-12, 10% bovine serum albumin (BSA, Sigma-Aldrich), 2.8 mM sodium pyruvate (Lonza), 2.56 mM L-glutamine, insulin-transferrin-sodium selenite media supplement (ITS, Lonza; 1 *μ*M insulin, 5.64 *μ*g/mL transferring, 32 nM selenium), and 100 IU/mL P/0.1 mg/mL S, 0.1 nM 3, 3′, 5-Triiodo-L-thyronine sodium salt (T_3_, Sigma-Aldrich)) was changed. The NRCs were cultured for one week.

#### 2.1.2. Culture and Differentiation of Human Embryonic Stem Cells

We used the H7 human embryonic stem cell line (WiCell, USA). H7 cells were cultured on top of mouse embryonic fibroblasts (MEFs, Millipore, France/USA) in KSR medium (Knockout DMEM (Invitrogen), 20% serum replacement (SR, Invitrogen), 2 mM GlutaMax (Invitrogen), 1% nonessential amino acids (NEAA, Lonza), 50 U/mL P/S, 0.1 mM 2-mercaptoethanol (Invitrogen), and 8 ng/mL basic fibroblast growth factor (bFGF, R&D Systems, USA)) to maintain their pluripotency [27]. The cells were enzymatically passaged once a week using type IV collagenase (1 mg/mL, Invitrogen).

Differentiation was induced by coculturing the stem cells with END-2 cells [28] in 0% KO-SR hES medium (Knockout DMEM, 2 mM GlutaMax, 1% NEAA, 50 U/mL P/S, 0.1 mM 2-mercaptoethanol) which was changed on days 5, 8, and 12. On day 15, the medium was replaced with 10% KO-SR hES medium (Knockout DMEM, 10% SR, 2 mM GlutaMax, 1% NEAA, 50 U/mL P/S, 0.1 mM 2-mercaptoethanol) that was replenished every third day thereafter. The first beating areas normally appeared after 14 days of differentiation. When the cells had been differentiated for 20 to 55 days, they were dissociated into single cells. Approximately the same amount (approximately 4,000) of cells was plated on each coating, on top of or inside the gels, and on control wells (0.1% gelatin (Sigma-Aldrich) coated commercial 24- or 48-well plates). The hESC-CMs were usually cultured for one week.

### 2.2. Cell Characterization

Cells were plated at equal numbers onto nanofiber hydrogels and control wells. The cells were observed daily using a phase-contrast microscope (Nikon Eclipse TS100, Nikon, Japan) and several qualitative parameters were scored to determine the suitability of nanofiber hydrogels for supporting cardiomyocyte culture. These parameters included cell attachment, spreading, morphology, viability, detachment, and beating. Staining for cardiomyocyte markers was used to evaluate the alignment and spreading of cardiomyocytes. Cell attachment was also evaluated quantitatively by counting troponin T positive cells. With NRCs, cell attachment was evaluated by measuring the confluency of troponin-positive cells/well, whereas with hESC-CMs all troponin-positive cells from every replicate were calculated.

#### 2.2.1. Viability

The LIVE/DEAD Viability/Cytotoxicity Kit for mammalian cells (Molecular Probes, Inc., Invitrogen), which contains calcein AM to stain live cells green and ethidium homodimer-1 to stain dead cells red, was used to assess viability. The stained cells were observed using phase contrast and fluorescence microscopy (Olympus IX51, Olympus, Japan) and photographed using an Olympus DP30BW camera (Olympus, Japan).

#### 2.2.2. Immunocytochemical Staining

The cells were first fixed with 4% paraformaldehyde (Fluka, Italy) at room temperature for 20 minutes and then blocked at room temperature for 45 minutes prior to labeling with primary antibodies (mouse monoclonal to myosin ventricular heavy chain alpha/beta (MHC) (1 : 100) (Chemicon Temecula, USA), mouse monoclonal to myosin-specific ventricle of the mammalian heart (MLC2v) (1 : 100) (Synaptic Systems, Germany), and goat polyclonal to cardiac troponin T (1 : 2000) (Abcam, UK)) at 4°C overnight. Cells were then labeled with secondary antibodies (Alexa 488 anti-mouse donkey (1 : 400, 1 : 800) (Invitrogen) or Alexa 568 anti-goat donkey (1 : 400, 1 : 800) (Invitrogen)) at room temperature for 2 hours. DAPI (4,6′*-*diamidino-2-phenylindole) was used to stain the nuclei. The cells were visually observed and photographed using the same equipment described for LIVE/DEAD staining.

### 2.3. Synthesis of Self-Assembling Nanofiber Hydrogels

**Figure 1:**
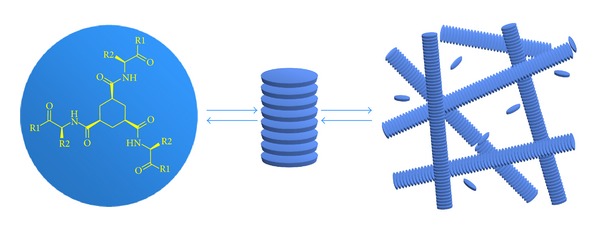
A schematic representation of gelation through self-assembly.

Self-assembling nanofiber hydrogels (Figure 1) were synthesized according to previously described methods [15, 16]. Self-assembling nanofiber hydrogels **1**–**6** were used in this study, and their structural formulas and fiber properties are listed in Table 1. 

#### 2.3.1. Cryogenic Transmission Electron Microscopy

Several microliters of the nanofiber suspensions were deposited on bare 700 lines/inch mesh copper grids. After excess liquid was blotted away, the grids were plunged quickly into liquid ethane. Frozen-hydrated specimens were mounted on a cryo-holder (Gatan, model 626) and observed using a Philips CM 120 electron microscope operated at 120 kV. Micrographs were recorded under low-dose conditions using a slow-scan CCD camera (Gatan, model 794).

#### 2.3.2. Preparation of 2D Nanofiber Coatings of pH-Sensitive Gelators (Nanofiber Coatings 1, 2)

A solution of 10 mg of the HCl salt (Boom B.V., The Netherlands) of the gelator in 3 mL of mQ water was prepared by gentle heating. The solution was neutralized by addition of 1 mL of 100 mM HEPES, pH 8 (Sigma-Aldrich). Aliquots of the 200 *μ*L neutralized solution were transferred to wells of a 24-well plate (SPL Life Sciences, Inc., the Republic of Korea). The solvent was evaporated overnight under ambient conditions to yield transparent to translucent coatings. The fibrous nature of the coatings was confirmed by optical microscopy (Motic AE31, China). Before use, the plates were sterilized for 5 minutes with UV light irradiation in a laminar flow cabinet. Nanofiber coating **1** was tested using both NRCs and hESC-CMs and nanofiber coating **2** was tested using hESC-CMs. 

#### 2.3.3. Preparation of HyA-Containing 2D Nanofiber Coatings of pH-Sensitive Gelators (Nanofiber Coatings **1** + HyA, **2** + HyA)

A solution of 10 mg of the HCl salt of the gelator in 2.6 mL mQ water was prepared by gentle heating. To this solution we added 0.4 mL of a 0.5% (w/v) solution of hyaluronic acid (Sigma-Aldrich) in mQ water. The resulting solution was neutralized by the addition of 1 mL of 100 mM HEPES, pH 8. Aliquots of the 200 *μ*L neutralized solution were transferred to the wells of a 24-well plate. The solvent was evaporated overnight under ambient conditions to yield transparent to translucent coatings. The fibrous nature of the coatings was confirmed by optical microscopy. Before use, the plates were sterilized for 5 minutes with UV light irradiation in a laminar flow cabinet. Nanofiber coatings **1** + HyA and **2** + HyA were tested using both NRCs and hESC-CMs.

#### 2.3.4. Preparation of 2D Coatings of Non-pH-Sensitive Gelators (Nanofiber Coatings and **3**, **4**, ** 5**, **6**)

A solution of 10 mg of the gelator in 16 mL of a 95 : 5 ethanol/water mixture was prepared by gentle heating. Aliquots of 750 *μ*L of the stock solution were transferred to the wells of a 24-well plate. The solvent was evaporated overnight under ambient conditions to yield transparent to translucent coatings. The fibrous nature of the coatings was confirmed by optical microscopy. Before use, plates were sterilized for 5 minutes with UV light irradiation in a laminar flow cabinet. Nanofiber coatings **3**, **5**, and **6** were tested using both NRCs and hESC-CMs and nanofiber coating **4** was tested using hESC-CMs.

#### 2.3.5. Preparation of 3D Nanofiber Hydrogels

Based on the results from the 2D experiments, the nanofiber coatings **1** + HyA and **4** were chosen for the 3D nanofiber hydrogel experiments. Nanofiber hydrogel **1** was included in the hESC-CM experiments to control for the effects of HyA addition. The 3D nanofiber hydrogels **1** + HyA and **4** were first studied using NRCs and then using hESC-CMs.

The gelators were dissolved in DMSO (Sigma-Aldrich) (**4**: 100–197 mg/mL) or 0.21 M HCl (**1**: 130 mg/mL). When using HyA in addition to the nanofiber hydrogel, the HyA was first diluted in medium (5 mg/mL). The gel stock and HyA solutions were then sterilized with UV light for 5 minutes. Finally, the gel stock solutions were diluted in medium (either with or without the cells) in a ratio of 1 : 9. If the medium did not contain cells, the cells were subsequently plated on top of the nanofiber hydrogels.

### 2.4. Statistics

Statistical significance for cell attachment was analyzed using The Kruskal-Wallis and Mann-Whitney tests.

## 3. Results

### 3.1. Nanofibers

The properties of the nanofiber hydrogels have been previously described in detail [15, 16]. Briefly, the nanofiber hydrogels are thermoreversible and their stability can be adjusted by adding amino-acid-based substituents. The substituents also affect the responsiveness of the hydrogel to pH changes [15], which results in hydrogels that are pH sensitive or non-pH sensitive (Table 1). The pH-sensitive nanofiber hydrogels are positively charged and the non-pH-sensitive hydrogels are neutral. The positively charged pH-sensitive hydrogels can be coassembled with negatively charged HyA by electrostatic interaction.

Nanofiber hydrogels have fibers thicknesses that range from nanometers to micrometers (Table 1) and they form fibrous gel networks [15, 16]. The fiber surfaces are either cationic (positively charged) or protic (protons on the surfaces that exchange with water) (Table 1).

### 3.2. Cryogenic Transmission Electron Microscopy

**Figure 2:**
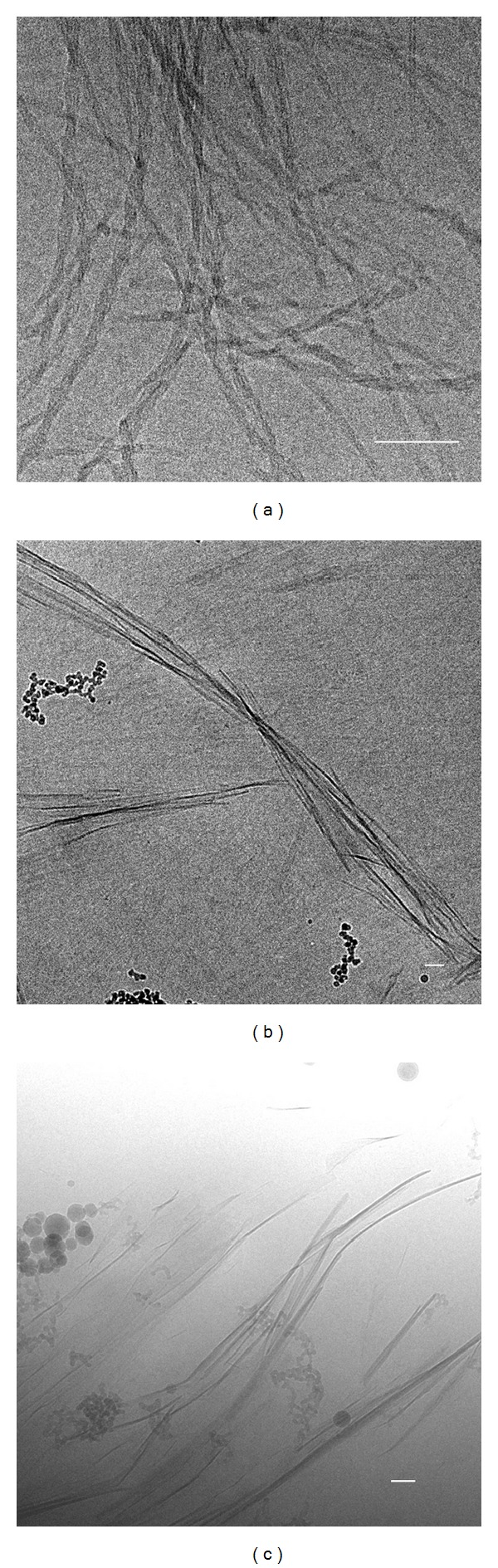
Cryo-TEM images of nanofiber hydrogels **3** (0.03 wt%, (a)), **4** (0.2 wt%, (b)), and **5** (0.05 wt%, (c)). Scale bars represent 100 nm.

Whereas gelators **1**, **3**, **4**, and **5** all produced very similar fiber surfaces with terminal hydrophilic alcohol groups reminiscent of polyethylene glycol, the self-assembly process for these four compounds resulted in fibers with pronounced differences in morphology. Thus, this series of compounds was studied to investigate the effects of similar surface chemistry but different morphology on cellular attachment. Cryogenic transmission electron microscopy (Cryo-TEM) was used to characterize the differences in fiber morphology for these four compounds. Compound **1** was previously reported to self-assemble into tubular fibers with a diameter of approximately 4.2 nm and a very homogeneous distribution [16].  Figure 2 shows that compound **3** self-assembles into bundles of fibers with a diameter of approximately 13 nm. Compound **4** self-assembles into ribbons of uniform thickness that are approximately 50–200 nm wide. Compound **5** self-assembles into sheets of uniform thickness with widths of 100 nm to 3 *μ*m.

### 3.3. 2D Nanofiber Coatings

**Figure 3:**
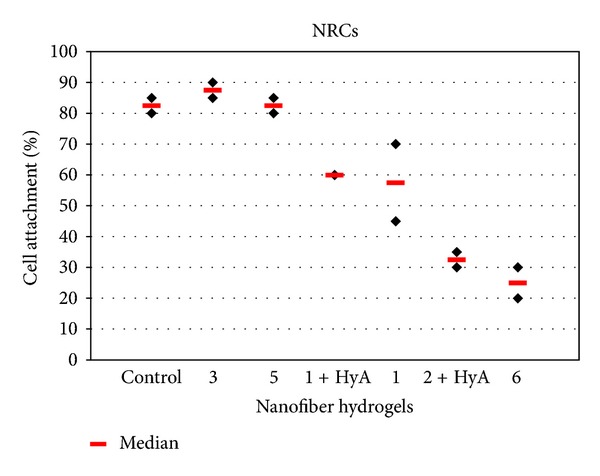
Cell attachment of NRCs was evaluated from troponin-T-positive cells.

**Figure 4:**
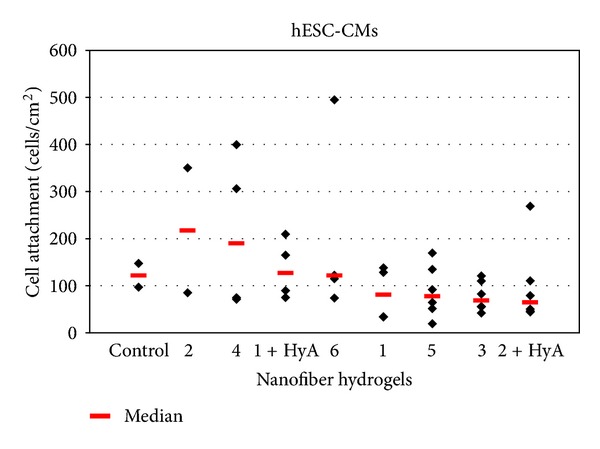
Cell attachment was determined by calculating the amount of troponin-T-positive hESC-CMs on each material. However, cell attachment was not the only criteria for optimal material and thus despite good attachment some nanofiber coatings were not optimal supporters for cardiomyocyte culture (for details, see Section 3).

The cells were cultured on the nanofiber coatings for seven days, after which they were stained with the LIVE/DEAD kit or fixed and stained with cardiac-specific antibodies (troponin T, MHC or MLC2v). The suitability of nanofiber coatings for cardiomyocyte culture was evaluated by observing cell attachment, spreading, morphology, viability, detachment, and beating in comparison to cells cultured on control surfaces (NRCs on untreated commercial well plates and hESC-CMs on 0.1% gelatin-coated commercial well plates). The results for the evaluation criteria for each material are summarized in Table 1. In addition, cell attachment was quantified (Figures 3 and 4), but no statistical significance was detected.

**Figure 5:**
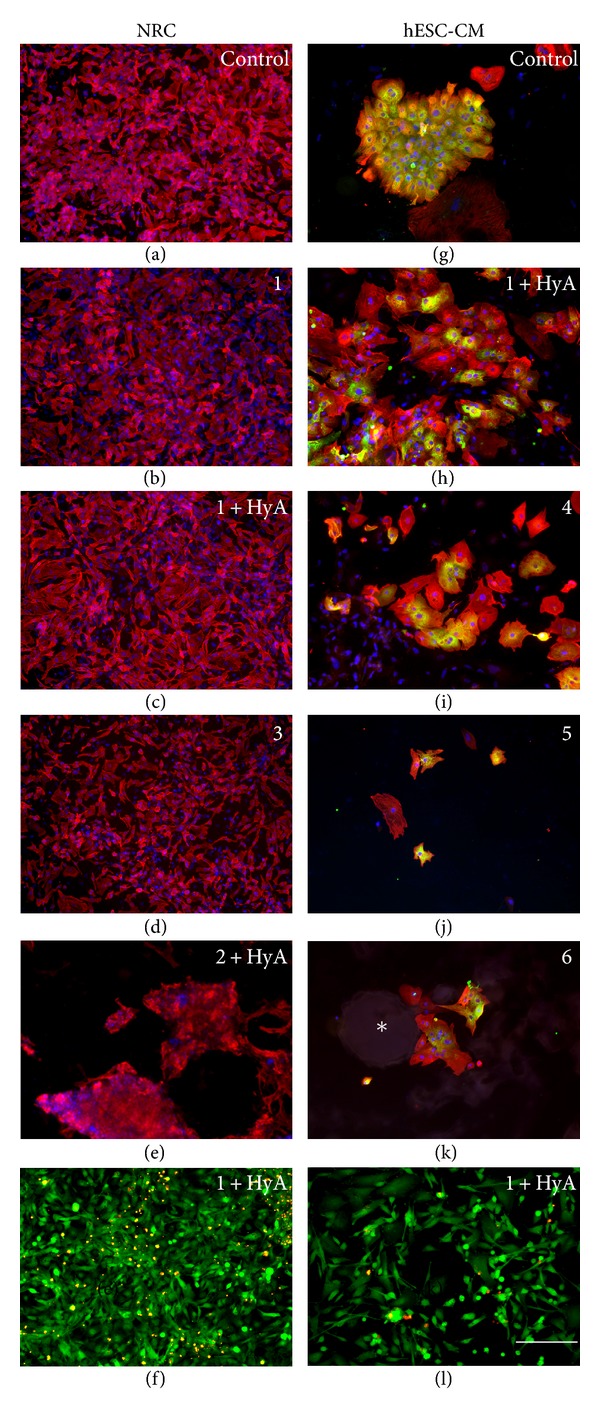
Cardiomyocytes on different nanofiber coatings stained positively using ((a)–(e), (g)–(k)) cardiac antibodies or ((f), (l)) LIVE/DEAD Kit. Red is ((a)–(e), (g)–(k)) troponin T, green is (g) MLC2v or ((h)–(k)) MHC, and blue is DAPI (nuclei). NRCs grew as well on top of nanofiber coatings (b) **1** (*n* = 2), (c) **1** + HyA (*n* = 2), and (d) **3** (*n* = 2) as on (a) the control (untreated commercial well plate, *n* = 2), whereas on (e) **2** + HyA (*n* = 2) they did not attach well and formed clusters. Cardiomyocytes differentiated from hESCs grew well on top of nanofiber coatings (h) **1** + HyA (*n* = 5) and (i) **4** (*n* = 4) and fairly well on nanofiber coating (j) **5** (*n* = 6) when compared to (g) the control (commercial well plate coated with 0.1% gelatin, *n* = 2). On nanofiber coating (k) **6** (*n* = 4), the cells did not grow on top of the coating, but they surrounded it. The round structure (∗) is a particle of the coating. The ratio between living and dead cells was approximately the same on every coating with both (f) NRCs and (l) hESC-CMs. The same scale bar (200 *μ*m) applies to every image.

The ratio between live and dead cells for both cell types was almost the same on every nanofiber coating. Approximately 70% of cells were alive and 30% were dead (Figures 5(f) and 5(l)).

#### 3.3.1. Neonatal Rat Cardiomyocytes

There were no major differences in cell growth among the nanofiber coatings when culturing NRCs. Nanofiber coatings **1** (*n* = 3, Figure 5(b)), **1** + HyA (*n* = 3, Figure 5(c)), **3** (*n* = 3, Figure 5(d)), and **5** (*n* = 3) supported the growth of the NRCs equally as well as the control surface (*n* = 3, Figure 5(a)). The cells spread evenly and their morphology was the same as the cells in the control wells. Most of the cells remained attached throughout the entire culture period (Figure 3). The beating rate and strength were similar between nanofiber coatings and control wells.

Nanofiber coatings **6** (*n* = 3) and **2** + HyA (*n* = 3, Figure 5(e)) did not support the growth of NRCs as well as the control surface and the cells did not attach properly on these coatings (Figure 3). Also the attached cells tended to detach over time. At day 7, most of the cells had detached and formed aggregates on the coatings (Figure 5(e)). Additionally, cells were not as evenly spread on these nanofiber coatings as on the control surface (Figure 5(a)).

#### 3.3.2. Human Embryonic Stem-Cell-Derived Cardiomyocytes

Hydrophilic nanofiber coatings **1** + HyA (*n* = 7, Figure 5(h)) and **4** (*n* = 4, Figure 5(i)) were best suited for supporting hESC-CMs. The cells attached well and they spread as evenly on the nanofiber surfaces as on the gelatin control surface (*n* = 3, Figure 5(g)). Nanofiber coating **1** + HyA supported the growth of hESC-CMs throughout the entire culture period of seven days. Nanofiber coating **4** also supported the growth and survival of the cells, although initially cells needed a few days to adapt to this coating. Once adapted, the cells spread evenly and exhibited regular beating.

Nanofiber coatings **5** (*n* = 7, Figure 5(j)) and **2** + HyA (*n* = 7) modestly supported hESC-CM growth. On nanofiber coating **5** (Figure 5(j)), there were fewer hESC-CMs attached and the attached cells were smaller than those on the control surface (Figure 5(g)). Nanofiber coating **2** + HyA did not perform well initially, but towards the end of the culture period, the hESC-CMs adapted and spread well on this surface.

Nanofiber coatings **1** (*n* = 7), **2** (*n* = 2), **3** (*n* = 5), and **6** (*n* = 5) did not support the growth of hESC-CMs. The cells either did not attach at all or they detached shortly after attachment. Some attached cells remained spherical and did not spread on the nanofiber coatings. On nanofiber coating **6,** the cells surrounded the nanofiber particles rather than growing on top of them (Figure 5(k)).

Nanofiber coatings **1** + HyA and **4** were evaluated as the best ones due to the cell attachment calculations (Figure 4) and other evaluated criteria (namely, cell spreading, morphology, viability, detachment, and beating; Table 1). Although nanofiber coating **2** had higher median cell attachment than **1** + HyA and **4**, it was classified as one of the least supportive coatings because the morphology of the cells was not the same as on the controls but spherical and they did not spread on the coating. In addition, there was high variation on cell attachment. Also nanofiber coatings **1**, **3**, and **6** were classified as the least supportive materials, not according to the amount of cells attached (Figure 4) but according to other evaluation criteria listed above (Table 1).

### 3.4. 3D Nanofiber Hydrogels

Nanofiber coatings **1** + HyA and **4** were chosen for the nanofiber hydrogel experiments because of their superior performance in the 2D coating experiments with hESC-CMs. Nanofiber coating **1** was also investigated as a control for the nanofiber hydrogel **1** + HyA. The purpose of the 3D experiments was to see how well the 2D results translated to 3D. The same evaluation parameters were used as in the 2D coating experiments. One additional parameter was used, that is, degradation of the hydrogels. LIVE/DEAD staining could not be used because of high background.

#### 3.4.1. Neonatal Rat Cardiomyocytes

NRCs grew well both on top of and inside the nanofiber hydrogels. Nanofiber hydrogel **1** + HyA (*n* = 3) performed well and was stable for the entire duration of the experiment (7 days). Nanofiber hydrogel **4** (*n* = 3) supported cell growth well but appeared to be better suited for short-term studies or for other applications, as the gels started to degrade a few days after the cells were plated. The degradation of nanofiber hydrogel **4** was remarkably faster than that of nanofiber hydrogel **1** + HyA.

#### 3.4.2. Human Embryonic Stem-Cell-Derived Cardiomyocytes

The three nanofiber hydrogels also supported the growth of hESC-CMs. All of the tested nanofiber hydrogels allowed the hESC-CMs to grow and beat on top of the gels as well as inside the gels; however, nanofiber hydrogel **4** (*n* = 3) degraded too rapidly for long-term use (this was also observed for NRCs). Nanofiber hydrogel **1** + HyA (*n* = 5) performed better than non-coassembled nanofiber hydrogel **1** (*n* = 3). In hydrogel **1** + HyA, the cells remained inside the gel, whereas, in non-coassembled nanofiber hydrogel **1**, the cells tended to migrate through the hydrogel to the bottom of the wells. In addition, nanofiber hydrogel **1** + HyA was more robust, as it remained intact for the entire 30-day duration of the experiment. Furthermore, this hydrogel was able to maintain the beating capability of the cells (see supporting data (video) in supplementary material available at doi:10.1155/2012/285678). 

## 4. Discussion

In this paper, we evaluated the suitability of six different self-assembling nanofiber hydrogels for attachment and growth of NRCs and hESC-CMs, first as 2D coatings and then as 3D gels. We examined nanofiber hydrogels that were pH sensitive, non-pH sensitive, hydrophilic, and hydrophobic. Hyaluronic acid was coassembled with the pH-sensitive hydrogels. Two hydrophilic synthetic nanofiber hydrogels were found to support human and rat cardiomyocytes in both 2D and 3D culture, thus providing an alternative platform for *in vitro* cardiac modeling.

Collagen has been extensively studied as a biomaterial for cardiac tissue engineering [8, 10, 11]. Although natural polymers such as collagen may be beneficial for cell attachment and differentiation, they often do not have the proper mechanical strength. Batch-to-batch variations [29] and possible contamination with animal compounds also raise concerns, especially when considering future clinical applications. In addition, for proper gelation of collagen, animal-derived materials such as Matrigel, chick embryo extract, horse serum, or extracellular matrix (ECM) from decellularized porcine hearts are added [10, 23]. In contrast, fully synthetic materials are homogenous, well defined, and have low batch-to-batch variation. Synthetic compounds can easily be modified using amino-acid-based substituents [15] and are thus considered to be reliable and customizable materials for *in vitro* and clinical applications. Furthermore, synthetic compounds can be produced without any animal products, which is desirable for clinical applications.

In our previous study [14], we showed that the growth of NRCs was best supported by natural collagen. Therefore, we wanted to continue our studies using a material with properties similar to those of natural collagen. Self-assembling nanofiber hydrogels are an emerging class of synthetic biomaterials that offer highly bioactive nanostructures that can be of interest for many biomedical applications [30, 31]. Structurally, self-assembling nanofiber hydrogels have a strong resemblance to natural collagen and their biocompatibility has been demonstrated [20]. Hence, these materials were potentially suitable for supporting the growth of cardiomyocytes, which was indeed shown in this study.

In our 2D coating experiments, all nanofiber coatings supported the growth and survival of NRCs. However, two of the nanofiber coatings supported only limited attachment and growth of NRCs: one was the hydrophobic non-pH sensitive nanofiber **6** and the other was the hydrophilic pH-sensitive nanofiber **2**. The best coatings for NRCs were the hydrophilic pH-sensitive nanofiber coating **1** with or without HyA and the hydrophilic non-pH sensitive nanofiber coatings **3** and **5**. According to these results, hydrophilicity is more effective at promoting cell attachment and growth than pH sensitivity. However, pH-sensitive nanofiber hydrogels are positively charged and are therefore expected to provide greater cell attachment because cells have negatively charged surfaces. In these experiments, pH sensitivity seemed to work well when combined with hydrophilicity.

For hESC-CMs, the differences in cell attachment and survival among 2D nanofiber coatings were more pronounced. The nanofiber **1** hydrogel with hydrophilic fiber surface provided the best attachment and growth support also for human cardiomyocytes. The addition of HyA to nanofiber coating **1** further improved cell attachment and survival and also provided the best support for cells in 3D. Hyaluronic acid is one of the components of the ECM. Therefore, it was presumed that HyA improved cell attachment [32] as well as cell proliferation and migration [33]. It would have been interesting to see how the addition of HyA to other nanofiber hydrogels affects cell attachment and survival, but it is possible to co-assemble negatively charged HyA only with positively charged pH-sensitive hydrogels.

The nanofiber hydrogel **2** (± HyA) did not support the growth of cardiomyocytes; only NRCs grew on this material to some extent. The fiber surfaces of this hydrogel are lysine-terminated and thus similar to polylysine surfaces in terms of chemistry. Polylysine is considered favorable in terms of cell attachment. The attachment mechanism of cells to polylysine has not been fully elucidated, but it is usually considered to involve an electrostatic interaction between anionic cell surfaces and the cationic polylysine surface [34]. Therefore, nanofiber hydrogel **2** surface was expected to enhance cell attachment more effectively than other surfaces, but this was not observed, which could be due to the more basic nature of nanofiber hydrogel **2** than polylysine. Nanofiber hydrogel **6** was also unsuitable for supporting cardiomyocytes; however, this hydrogel has a very hydrophobic fiber surface and thus these results were expected.

Nanofiber hydrogels **1**, **3**, **4**, and **5** all have very similar fiber surfaces, but the fiber thicknesses vary, which allowed us to examine the effects of similar surface chemistries and different fiber morphologies on cellular attachment. We varied the thickness of the fibers: **1** (4.2 nm), **3** (13 nm), **4** (50–200 nm), and **5** (100 nm–3 *μ*m). For comparison, collagen fibers are 1–20 *μ*m thick and elastin fibers are 0.2–1.5 *μ*m thick [35]. Nanofibers in hydrogel **5** formed sheets which thicknesses are similar to the fiber thicknesses of collagen and elastin. Nanofiber hydrogel **1** had the thinnest fibers. In our study, nanofiber hydrogel **1** (+ HyA) had the best performance. The second thickest nanofiber hydrogel (nanofiber hydrogel **4**) had fiber sheet thicknesses of 50–200 nm and also performed well with hESC-CMs. Among nanofiber hydrogels with protic fiber surfaces, fiber thickness had less influence on NRC attachment than HyA addition. However, the fiber dimensions and the addition of HyA both affected hESC-CMs attachment.

Some differences in the results between NRCs and hESC-CMs were observed, especially in 2D experiments. It has been reviewed in previous experiments that hESC-CMs are more sensitive to surrounding biomaterial than other cell types [36]. NRCs are more robust and also in our experiments grew better on several nanofiber hydrogels than hESC-CMs. Shapira-Schweitzer et al. also showed that NRCs are easier to handle, maturate faster, and contract more effectively than hESC-CMs [22]. Nevertheless, NRCs can be used as a model for human cells for preliminary experiments, for example, to screen different biomaterials. This was also demonstrated in our study as hESC-CMs did not grow well on any of the materials where NRCs did not grow.

When evaluating nanofiber materials as in the present study, it is important to note that both NRC- and hESC-derived beating areas contain both cardiomyocytes and noncardiomyocytes. Consequently, when the beating areas are dissociated, there is always a mixture of cell types. As a result, it is currently not possible to obtain pure populations of human cardiomyocytes for testing. However, the other cell types existing in the beating areas, such as fibroblasts, have been shown to support cardiomyocyte growth and functionality [37], which suggests that the mixed population of cells in our cultures is beneficial. Additionally, the presence of other cell types in cardiac grafts (i.e., fibroblasts and endothelial cells (ECs)), has been demonstrated to improve the vascularization and function of grafts *in vivo* [38].

As cells grow in 3D *in vivo*, we wanted to test whether nanofibers in 3D hydrogels would support cell growth better than on 2D coatings. However, our results did not support this hypothesis, possibly because of problems with the gelation process. The optimal amount of cells needed for formation of 3D cell structures is also not known. In 3D hydrogel experiments, some variation from well to well was observed, mainly because gelation occurred rapidly resulting in heterogeneous hydrogels. Consequently, in softer parts of the hydrogels, some of the cells migrated to the bottom of the wells rather than staying attached to the hydrogel matrix. The cells that stayed attached to the hydrogels grew well and retained their beating capability. The heterogeneous nature of the hydrogels sometimes limited the visualization of the cells. There were no major differences in the growth pattern of cells inside or on top of the different nanofiber hydrogels for either of the cell types.

Not many 3D biomaterial studies have been performed using hESC-CMs. Our study is the first to demonstrate the growth of hESC-CMs in 3D self-assembling nanofiber hydrogels. The first 3D vascularized human cardiac tissues were created by combining hESC-CMs, ECs, and fibroblasts with PLLA(50%)/PLGA(50%) biodegradable scaffolds [24]. Transplantation of hESC-CMs in alginate scaffolds into infarcted heart tissue has also been described; however, this treatment did not promote myogenic differentiation or organization of the implanted cells [39]. Furthermore, hESC-CM function and maturation within PEGylated fibrinogen (PF) hydrogels has been shown. The responsiveness of these cells to cardiac drugs demonstrated the potential to use this system for *in vitro* drug screening [22]. The same group showed that codelivery of PF matrix with hESC-CMs into infarcted areas provided additional therapeutic effects and prevented unfavorable postinfarction cardiac remodeling [40]. Madden et al. showed that hESC-CMs seeded into microtemplated poly(2-hydroxyethyl methacrylate-co-methacrylic acid) (pHEMA-co-MAA) hydrogels cultured for 2 weeks could reach adult heart density *in vitro*. Additionally, acellular scaffolds implanted in rats enhanced angiogenesis [41]. In one study, porcine heart ECM and collagen I were used to induce cardiac differentiation of hESCs. Hydrogels with different ratios of ECM and collagen were prepared and cardiomyocyte maturation and contraction were evaluated. Hydrogels with a higher ECM content promoted cardiac maturation [23]. Human engineered heart tissue (hEHT) has also been developed from hESC-CMs and fibrinogen; it forms a dense network that responds to chronotropic compounds [42].

Based on our study, self-assembling nanofiber hydrogels can be modified to obtain beneficial features that support the growth of cardiomyocytes. To further improve the performance of these synthetic cell-supporting structures, the gelation procedure should be optimized to allow more homogeneous formation of the hydrogels. Another possibility that we are now investigating is decoration of the self-assembling nanofiber hydrogels with functional groups (e.g., the RGD peptide sequence) to improve cell adhesion. Finally, because a main application for 3D cardiac tissue is drug discovery and testing, the possibility of measuring signals from hESC-CMs in a 3D hydrogel using a microelectrode array (MEA) is under investigation. The suitability of MEA platforms for measuring drug responses of hESC-CMs has been shown in 2D culture [28, 43, 44].

## 5. Conclusions

In our previous study, we compared different natural and synthetic biomaterials for cardiomyocyte culture. Collagen type I best supported the growth of cardiomyocytes. However, as a natural material, collagen has batch-to-batch variations. We therefore decided to investigate a synthetic material similar to collagen. In this study, neonatal rat cardiomyocytes and human embryonic stem-cell-derived cardiomyocytes were grown on different synthetic self-assembling nanofiber hydrogels. The pH-sensitive nanofiber hydrogel with hydrophilic and protic fiber surfaces and coassembled with hyaluronic acid best supported the growth of rat and human cardiomyocytes. These nanofiber hydrogels are promising materials for the development of future cardiac tissue models.

## Supplementary Material

Beating hESC-CMs on nanofiber hydrogel 1(number should be in bold)*+*HyA.

## Figures and Tables

**Table 1a:** (a)

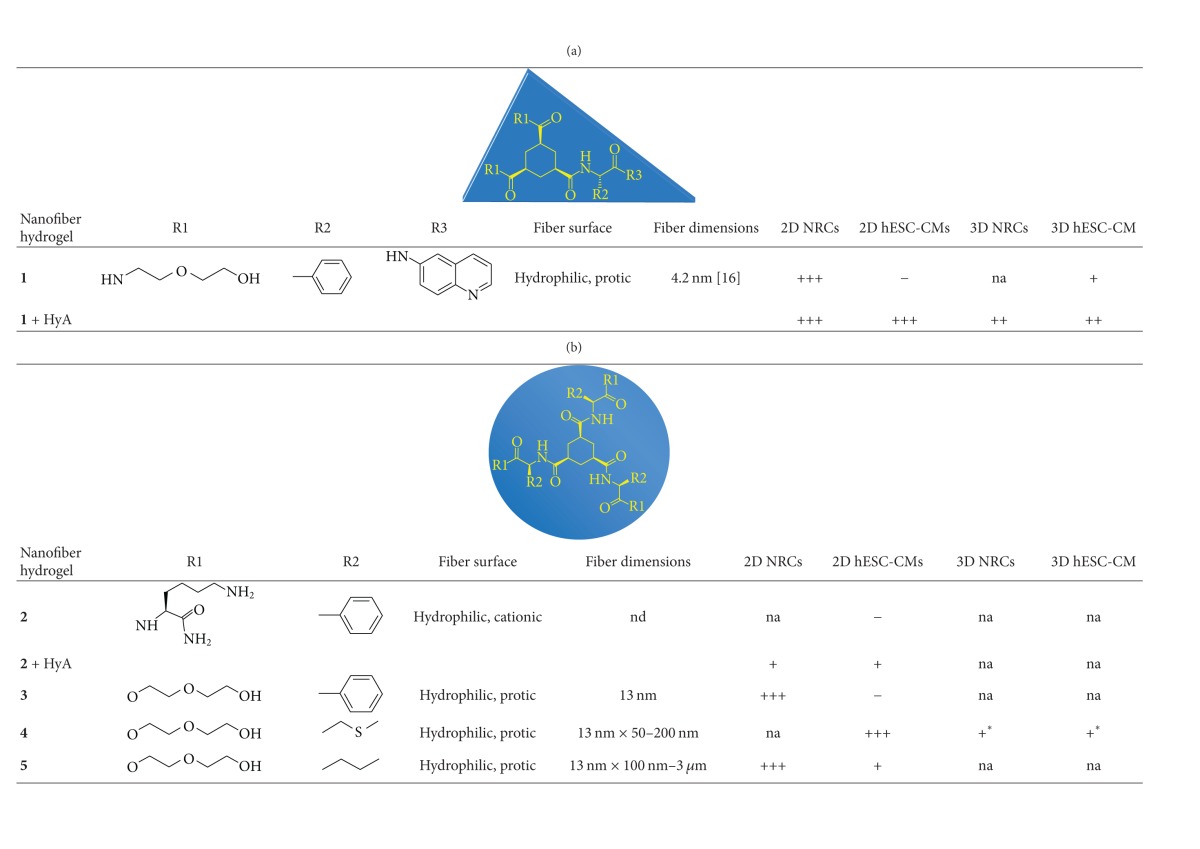

**Table 1b:** (b)

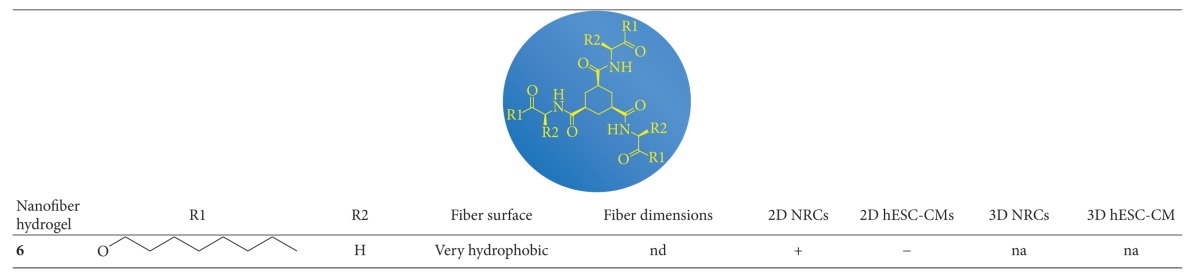

nd: not defined.

na: not analyzed.

+++: preferable material with as good performance as the control.

++: suitable material with lower performance than the control.

+: sufficient material with inferior performance than the control.

−: unsuitable material because cells did not attach.

*The nanofiber hydrogel degraded too fast.
